# The role of standard automated perimetry and newer functional methods for glaucoma diagnosis and follow-up

**DOI:** 10.4103/0301-4738.73694

**Published:** 2011-01

**Authors:** Luciana M Alencar, Felipe A Medeiros

**Affiliations:** Department of Ophthalmology, Hamilton Glaucoma Center, University of California, San Diego, CA, USA

**Keywords:** Frequency doubling perimetry, guided progression analysis, short-wavelength automated perimetry, standard automated perimetry, Sweedish interactive thresholding algorithm

## Abstract

Automated perimetry has become the mainstream for assessment of functional glaucomatous loss and progressive damage. Recent improvements with the Swedish interactive thresholding algorithm (SITA) strategy and the guided progression analysis (GPA) have further settled standard achromatic perimetry (SAP) as the preferred method for diagnosis and follow-up of functional loss. Although SAP is still considered the gold standard, function-specific perimetry may offer advantages for early diagnosis. Frequency doubling technology (FDT) and short-wavelength automated perimetry (SWAP) have been shown to be helpful, especially when SAP is normal and there is a suspicion of glaucoma. Studies using rarebit perimetry have also shown promising results. Studies have observed that each test identifies a different subset of eyes, and combining the tests may improve sensitivity. Nevertheless, the more sophisticated analyses do not reduce the importance of a correct interpretation of the test results.

Automated perimetry has become the mainstream for assessment of functional glaucomatous loss and progressive damage. For the past several years, several different perimeters and test strategies have been developed, but none has had more studies and has been more used than standard white-on-white perimetry with the Humphrey Field Analyzer (HFA; Carl-Zeiss Meditec, Dublin, CA, USA). Recent improvements with the Swedish interactive thresholding algorithm (SITA) strategy and the guided progression analysis (GPA) have further settled it as the preferred method for diagnosis and follow-up of glaucomatous functional loss. However, the more sophisticated analysis does not reduce the importance of a correct interpretation of the test results. Fig. 1 shows the printout of a SITA 24-2 test. The strategy used and size of stimulus can be found in the upper portion of the printout, along with patient identification, age, pupil size, visual acuity and near correction used [Fig. 1A]. All this information is relevant and should be taken in consideration when assessing the test results. Typically, a size III stimulus is used unless the visual acuity of the patient is below 20/200, when a size V may be needed. The first two graphs represent the numerical display of the threshold values and its corresponding gray scale map [Fig. 1B]. These plots represent raw threshold values, which are not adjusted for age or diffuse loss. Even though the gray scale map seems the easiest to evaluate, it is also less reliable. Threshold values are grouped in 5 dB increments and therefore different shades may represent differences from 1 to 9 dB. In addition, elderly patients and those with cataracts will always present darker shades that do not necessarily represent glaucomatous damage. The two most important plots are the total and the pattern deviation probability plots. The total deviation values and corresponding probability plot [Fig. 1C] represent the differences between the thresholds obtained for each test point against what is expected among normals of the same age. Defects noted in this plot are not specific for glaucoma, especially when diffuse. The next step is to look at the pattern deviation values and the corresponding probability plot [Fig. 1D]. The values in this plot have been adjusted to remove the effects of diffuse loss and bring to light any localized defects. The pattern deviation plot is very useful to distinguish the effects of cataract from glaucomatous damage on the visual fields. It is important to remember, however, that in more advanced stages of the glaucoma disease one may find such severely compromised visual fields that no localized depressions are observed and the pattern deviation plot may appear almost normal.

**Figure 1:**
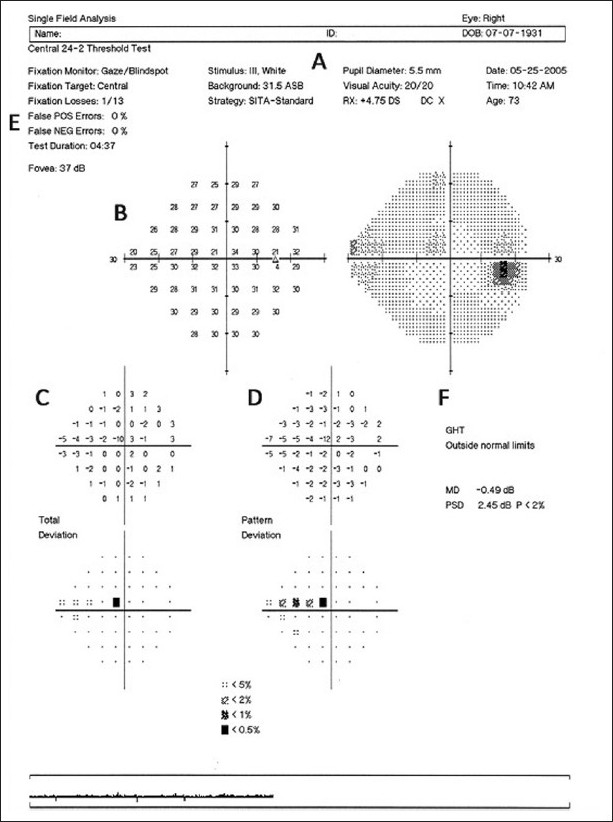
Example of the printout obtained for a white-on-white test using the SAP 24-2 SITA algorithm. (A) Identification of the patient, the stimulus used, and the test algorithm. (B) Numerical display of the threshold values and its corresponding gray scale map. (C) Total deviation values and corresponding probability plot. (D) Pattern deviation values and corresponding probability plot. (E) Reliability indices. (F) GHT and global indices

In every exam, it is crucial to check the reliability indices. One can only rely on the results of a visual field exam if the patient was able to follow the instructions and correctly perform the test. Even though the analysis is fully automated, the exam still relies upon the cooperation of the patient. There are three reliability indices reported on the upper left of every exam [Fig. 1E]. The rate of fixation losses represents the number of times the patient lost fixation to the central target and can be high with distraction and fatigue. False positive rates represent responses without an associated stimulus, usually due to lack of understanding of the test or anxiety from the patient. Exams with a high number of false positives are almost invariably unreliable. False negatives represent points in which the patient failed to respond to an above-threshold stimulus. It can be a consequence of inattention from the patient. However, in more advanced cases, it may reflect the larger fluctuation in response on the borders of pre-existing scotomas. Therefore, it is unexpected to find high false negative rates in normal exams or in early stages of disease, but it is not unusual in more advanced cases. As a general rule, for the three indices, rates below 33% are usually adequate, although rates of false positives should be preferably below 15%.[1]

The glaucoma hemifield test (GHT) is an additional analysis based on the asymmetry in severity observed in glaucoma between both hemifields [Fig. 1F]. It represents the comparison of five zones in the superior hemifield with the respective mirror-image zones in the inferior hemifield. Depending on the presence of asymmetry across the midline, possible results are *Outside Normal Limits, Borderline* and *Within Normal Limits*. In addition, the GHT may report *General Reduction of Sensitivity* or *Abnormally High Sensitivity*, for diffuse effects. Lastly, we see at the bottom right of the printout two global indices used to summarize the information from the total deviation and the pattern deviation plots [Fig. 1F]. The mean deviation (MD) value summarizes the results from the total deviation plot, and the more affected the visual field, the lower the MD. Similar to the total deviation values, the MD is susceptible to the effects of cataract and other causes of diffuse sensitivity loss. The pattern standard deviation (PSD) summarizes the results from the pattern deviation plot, and the presence of focal defects is reflected by an increase in the PSD value. Alternative algorithms have been studied, such as changing the threshold significance on probability plots.[2] However, these are still not available to clinicians and need further validation.

As visual field testing is a subjective examination, variable responses may be obtained each time the test is performed or even during the same test. This variability has been the biggest drawback of visual field assessment, as it may greatly confound interpretation of the test. Fluctuation varies among patients and among sectors in the same visual field, and usually increases with severity of disease. Any abnormalities in a visual field test should be confirmed in subsequent tests.

Irreversible visual field defects are the final common feature of glaucomatous damage to the retinal ganglion cells (RGC), and for many years, functional evaluation of these cells relied solely on white-on-white standard automated perimetry (SAP). However, although SAP remains the most commonly performed method of visual field assessment in glaucoma, histological and clinical studies have shown that in many cases visual field defects on SAP are detectable only when a substantial number of ganglion cells have been lost.[3,4] Several factors seem to be related to the relative insensitivity of SAP to early RGC damage, including the variability of the test and the considerable redundancy of the human visual system. Whereas light detection can be perceived by almost all RGCs, more specific features, such as contrast sensitivity, movement perception and color vision, are encoded by specific subsets of these cells. When one single pathway is isolated, a deficit may be manifest even when a small proportion of cells are affected because even if other cell types are still functioning in a given retinal area, they are unable to detect that specific stimulus. Frequency doubling technology (FDT) and short-wavelength automated perimetry (SWAP) have been shown to be helpful, especially when SAP is within normal limits and there is a suspicion of glaucomatous damage. Isolating subsets is only one of the several theoretical alternatives for an earlier diagnosis. More recently, studies using rarebit perimetry, which evaluates the density of coverage instead of thresholds per area, have also shown promising results.

The FDT perimetry determines the contrast sensitivity for detecting the frequency doubling stimulus. The frequency doubling illusion phenomenon was thought to be mediated by a subset of magnocellular RGCs.[5] However, recent research has suggested the origins of the response to be most likely cortical.[6–8] The FDT perimeter is portable and considerably easier to use for both the technician and the patient; the exam is faster than SAP and is more resistant to blurring effects. The FDT Matrix (Carl-Zeiss Meditec) is the latest commercially available version and offers a new additional testing program along with the same tests provided by the previous versions of this technology. In the new testing protocol, the FDT Matrix utilizes grating targets smaller than the original FDT to enable standard 24-2 and 30-2 test patterns, which look identical to those in SAP. Similar to conventional perimetry, the test gives both raw sensitivity values and probability plots [Fig. 2]. After comparing the results with age-matched normal individuals from the internal database, a statistical analysis package provides the total and pattern deviation plots, and the global indices MD and PSD. Reliability is also measured and shown with similar indices as SAP: fixation loss, false-positive, and false-negative. Several independent studies have shown that FDT has high sensitivity and specificity to discriminate glaucomatous patients from normal subjects, and that its results are predictive of future onset and location of functional loss assessed by SAP in glaucoma suspects.[9–12] After adjustment for other risk factors, a patient with an abnormal FDT test at baseline was threefold more likely to develop a confirmed abnormal SAP visual field during follow-up than a patient with a normal FDT.[12] These findings have also been confirmed by prospective studies.[13,14] Using the 24-2 Matrix, a recent study has shown that it performed significantly better than SAP SITA for detection of early glaucomatous damage, whereas in patients with more severe disease they observed no differences between their performances.[15] The FDT Matrix is a promising instrument for providing early diagnosis of glaucomatous functional loss and is especially helpful for subjects unable to perform SAP.[15–18]

**Figure 2:**
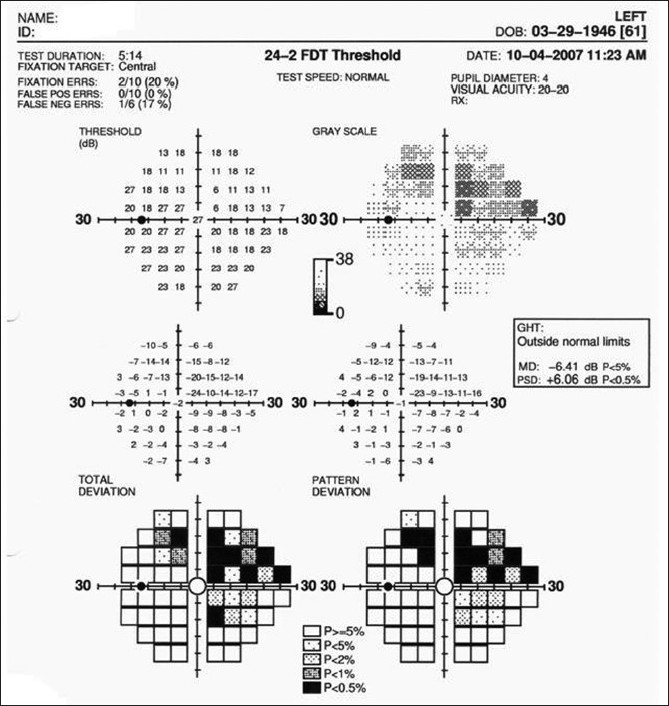
Example of the printout obtained with the FDT 24-2 Matrix. The reliability indices, threshold and probability plots, global indices and GHT are similar to those observed with standard achromatic perimetry

The SWAP, on the other hand, isolates the short-wavelength-sensitive pathway and differs from conventional perimetry for its narrow-band blue-light stimulus and the yellow background illumination. The software can be installed on HFA perimeters and, similar to SAP, in its most current version uses SITA strategy that reduces the overall duration of the test.[19] A statistical package compares the results with age-related normal subjects and provides likewise numerical and probability plots [Fig. 3]. The grayscale map on SWAP printouts, however, can be misleading because of the reduced visual perception of blue cones, resulting in a darker map even for normal subjects. The total and pattern deviation plots, the GHT and the global indices should be interpreted in the same way as for SAP. Overall, the interpretation of the results of SWAP testing should follow the same principles as for conventional achromatic perimetry. There is evidence indicating that SWAP is more sensitive than SAP (notably Full-threshold SAP) for detection of early functional deficits due to glaucoma.[20–22] Longitudinal studies showed that SWAP defects may occur 3–5 years before abnormalities are seen on Full-threshold SAP, and that they are predictive of both the onset and location of future SAP defects.[23–25] SWAP has traditionally been indicated as a test to evaluate visual function in glaucoma suspects, especially younger ones. The yellow tone of some initial nuclear cataracts may act as a blue filter and cause a significant diffuse depression of sensitivity.[26] Therefore, any diffuse depression of sensitivity seen on SWAP testing should be interpreted with caution.[19,23,25] Another important limitation of the SWAP Full-threshold strategy was the longer duration of the test and the limited dynamic range. Although these limitations have largely been overcome with the introduction of the SWAP SITA, the yellow background and the blue spot target of SWAP are still relatively more difficult to recognize than the white-on-white test, which increase patient fatigue and discomfort during the test. In addition, in advanced cases, the patient may not recognize even the brightest target; therefore, because of the narrow dynamic range, the test may not be sensitive enough to monitor progression in more advanced cases.

**Figure 3:**
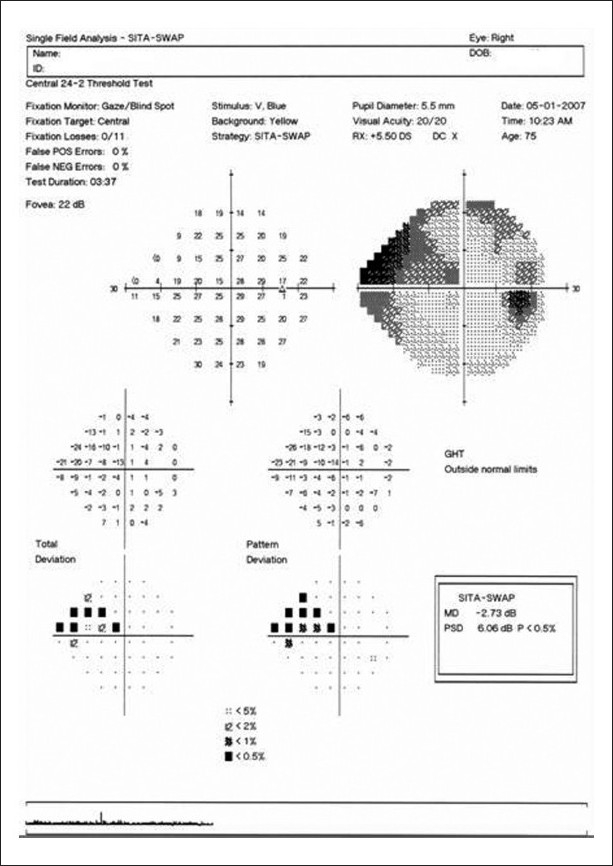
Example of the printout obtained with the Humphrey perimeter for a SWAP test using the SITA algorithm. The reliability indices, threshold and probability plots, global indices and GHT are similar to those observed with standard achromatic perimetry

Rarebit perimetry (or microdot perimetry) is a new technique that uses a regular computer LCD monitor to present very small stimuli (individual dots) on a dark background. The patient has to indicate for each presentation whether one or two dots were observed. Rarebit perimetry was not designed to isolate any specific type of RGC and there is no hill of vision. Instead of measuring thresholds for light sensitivity, it uses the microdots to evaluate the density of coverage within the central 30° of the visual field. Defects are seen as clusters of micro defects in the integrity of the system – as micro holes in a flat surface. Such micro defects aggregate in increased numbers within established visual field defects, and the deeper the defect, the larger the density of the micro holes. Rarebit visual field defects show spatial distributions similar to those of SAP, but defects containing sloping borders tend to appear larger in rarebit perimetry. It is a very recent technology but some studies have shown promising results.[27–29]

Few studies have compared the performance of the different visual function-specific tests in the same population, and most have used the previous generations of these instruments.[30] It is interesting to note that in most of these studies, each test identified a different subset of eyes, and that combining the tests improved sensitivity with only a slight reduction in specificity.[30] Although SAP is still considered the gold standard for functional evaluation in glaucoma, function-specific perimetric tests may offer several advantages for early diagnosis of functional loss but should not be done at the expenses of SAP. Hardware and software upgrades have overcome several limitations of the first generations of these instruments, but prospective longitudinal studies are still necessary in order to provide guidelines for clinicians on how to best incorporate the results from these new instruments into clinical practice.

Most frequently, progression is identified as deepening of a preexisting scotoma, along with enlargement of the defect. Diffuse sensitivity loss may also represent glaucoma progression, although it is usually accompanied by new localized defects or worsening of previous defects. Progressive diffuse loss that is isolated should always raise the suspicion of cataract progression or other media opacities. Before confirming the existence of a new or progressive visual field defect, it is important to demonstrate that the defect is repeatable on subsequent visual fields. This is one of the most important aspects of evaluating visual field progression in glaucoma. A few scattered points showing significant change are rather common in non-progressing patients. Variable responses that do not reflect further damage will vary in location and pattern in consecutive tests, whereas a true defect reflecting loss of RGC should repeat itself. Data from several clinical trials have suggested that change needs to be repeatable, with a defect of the same type in the same general location, in three consecutive exams before progression can be confirmed.[31–34]

There are two main approaches to analyze progression. The first is to compare the current examination with an older one (the baseline). If the results are significantly worse on the follow-up examination, it indicates progression. This is called event-based analysis as it looks for defects (events) on the current examination that were not present before. In the second approach, instead of comparing only a few tests, one looks for progressive change by analyzing all the tests available in a specific period of time. This is called trend-based analysis as change is observed as a trend in the values plotted over time, and significant deterioration can be assessed by observing the slope or decline of the regression line. Besides evaluating whether progression has occurred, trend analysis also allows the estimation of the rate of progression. It is well known that some patients decline faster than others, and estimating each individual’s rate of progression is helpful in evaluating response to therapy and determining how aggressive treatment should be.

Different analytical tools have been developed to assist clinicians in identifying visual field progression. The most general and simple way used the MD index plotted against time. Any significant decline was indicative of progressive deterioration. However, even though deterioration on the MD may represent glaucomatous progression, it may also represent progressing cataract or other media opacities. Conversely, cataract surgery in a glaucomatous patient may mask progression and even lead to improvement in MD values. Another analytical tool incorporated on previous versions of the Humphrey perimeters was the glaucoma change probability (GCP), which performed individual comparisons of each point at the follow-up examination with a set of baseline fields. Even though it performed a more individualized analysis of the sectors in the visual field, it was based on the total deviation plot and was still affected by diffuse media opacities such as cataract. The GPA software was developed to overcome most of these limitations. Both the GCP and the GPA are event-based analyses, but the GPA has several potential advantages when compared to the GCP. The GPA is based on the pattern deviation plots, as opposed to the total deviation plots used by the GCP. In addition, the GPA runs on SITA tests, but also accepts Full-threshold tests (the GCP does not), which is convenient for patients whose follow-up started with full-threshold perimetry. Detection of new or progressing visual field defects is performed by comparison to the baseline; therefore, it is critical to have reliable baseline exams. The software automatically selects the first two available exams as the baseline tests. However, one can easily override this selection to a more suitable time-point (e.g., change in therapy after progression) or to avoid initial learning effects (which could reduce the sensitivity to detect progression). The GPA software then compares each follow-up test to the average of the baseline tests. It identifies points that show change greater than the expected variability (at the 95% significance level), as determined by previous studies with stable glaucoma patients. If significant change is detected in at least three points, and repeated in the same points for two consecutive follow-up tests, then the GPA software will flag the last exam as *Possible Progression*. If the same three or more points have significant change detected and repeated in three consecutive follow-up tests, the GPA software will flag the last exam as *Likely Progression*.

The most recent version of the HFA also provides the visual field index (VFI) and VFI progression plot. The VFI is a newly developed index that is proposed to better evaluate the rate of progression with SAP. The aim of this analysis is mainly to provide valuable information on the rate of deterioration. The VFI is calculated as the percentage of normal visual field, after adjustment for age. Therefore, a VFI of 100% represents a completely normal visual field, while a VFI of 0% represents a perimetrically blind visual field. The VFI is shown on the GPA printout both as a percentage value for each individual exam and as a trend analysis, plotted against age [Fig. 4]. While the MD is based only on the total deviation map, and thus can be largely affected by cataract or other media opacities, the VFI is based both on the pattern deviation probability map, for the identification of possibly progressing points, and on the total deviation map, used for the actual calculation of change.[35] In addition, the algorithm gives more weight to central points, which have higher impact on the patient’s quality of vision. The final VFI score is the mean of all weighted scores. A recent study showed that the new summary index performed similarly to the MD for patients without cataract.[35] For glaucoma patients with worsening cataract, however, when compared to the MD, the VFI showed a slower rate of progression, which supposedly would be a more accurate representation, having reduced the effects of the cataract on the test. Conversely, for glaucoma patients who had cataract surgery during follow-up, the VFI showed a higher rate of progression compared to the MD. While the improvement in media clarity masked glaucoma progression assessed by the MD, this did not happen with the VFI. The VFI also provides an estimate of additional visual field loss for up to 5 years, considering that the same rate of progression is maintained. This is valuable for the clinician as it estimates the number of years that a specific patient has before advancing to a perimetrically blind visual field, if no further action is taken to improve control of the disease.

**Figure 4:**
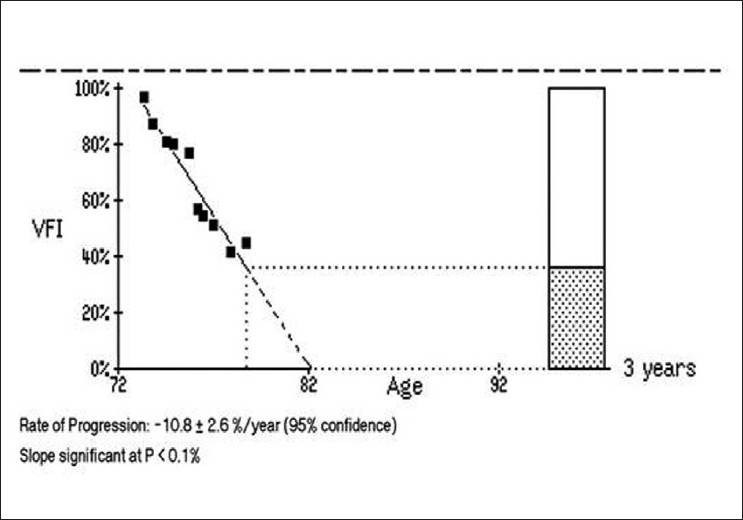
Example of the visual field index (VFI) plot. The solid line represents the estimated slope including all the exams. The dashed line represents the estimated slope for the following three years, considering that the same rate of progression is maintained. The rate of progression and slope significance are given along with the plot

## References

[1] Kelly DH (1981). Nonlinear visual responses to flickering sinusoidal gratings. J Opt Soc Am.

[2] Quaid PT, Simpson T, Flanagan JG (2004). Monocular and dichoptic masking effects on the frequency doubling illusion. Vision Res.

[3] Anderson RS (2006). The psychophysics of glaucoma: improving the structure/function relationship. Prog Retin Eye Res.

[4] White AJ, Sun H, Swanson WH, Lee BB (2002). An examination of physiological mechanisms underlying the frequency-doubling illusion. Invest Ophthalmol Vis Sci.

[5] Johnson CA, Keltner JL, Cello KE, Edwards M, Kass MA, Gordon MO (2002). Baseline visual field characteristics in the ocular hypertension treatment study. Ophthalmology.

[6] Wall M, Johnson CA, Kardon RH, Crabb DP (2009). Use of a continuous probability scale to display visual field damage. Arch Ophthalmol.

[7] Harwerth RS, Carter-Dawson L, Shen F, Smith EL, Crawford ML (1999). Ganglion cell losses underlying visual field defects from experimental glaucoma. Invest Ophthalmol Vis Sci.

[8] Kerrigan-Baumrind LA, Quigley HA, Pease ME, Kerrigan DF, Mitchell RS (2000). Number of ganglion cells in glaucoma eyes compared with threshold visual field tests in the same persons. Invest Ophthalmol Vis Sci.

[9] Johnson CA, Samuels SJ (1997). Screening for glaucomatous visual field loss with frequency-doubling perimetry. Invest Ophthalmol Vis Sci.

[10] Cello KE, Nelson-Quigg JM, Johnson CA (2000). Frequency doubling technology perimetry for detection of glaucomatous visual field loss. Am J Ophthalmol.

[11] Burnstein Y, Ellish NJ, Magbalon M, Higginbotham EJ (2000). Comparison of frequency doubling perimetry with humphrey visual field analysis in a glaucoma practice. Am J Ophthalmol.

[12] Medeiros FA, Sample PA, Weinreb RN (2004). Frequency doubling technology perimetry abnormalities as predictors of glaucomatous visual field loss. Am J Ophthalmol.

[13] Landers JA, Goldberg I, Graham SL (2003). Detection of early visual field loss in glaucoma using frequency-doubling perimetry and short-wavelength automated perimetry. Arch Ophthalmol.

[14] Landers J, Sharma A, Goldberg I, Graham S (2006). A comparison of diagnostic protocols for interpretation of frequency doubling perimetry visual fields in glaucoma. J Glaucoma.

[15] Medeiros FA, Sample PA, Zangwill LM, Liebmann JM, Girkin CA, Weinreb RN (2006). A statistical approach to the evaluation of covariate effects on the receiver operating characteristic curves of diagnostic tests in glaucoma. Invest Ophthalmol Vis Sci.

[16] Patel A, Wollstein G, Ishikawa H, Schuman JS (2007). Comparison of visual field defects using matrix perimetry and standard achromatic perimetry. Ophthalmology.

[17] Spry PG, Hussin HM, Sparrow JM (2005). Clinical evaluation of frequency doubling technology perimetry using the Humphrey Matrix 24-2 threshold strategy. Br J Ophthalmol.

[18] Sakata LM, Deleon-Ortega J, Arthur SN, Monheit BE, Girkin CA (2007). Detecting visual function abnormalities using the Swedish interactive threshold algorithm and matrix perimetry in eyes with glaucomatous appearance of the optic disc. Arch Ophthalmol.

[19] Moss ID, Wild JM, Whitaker DJ (1995). The influence of age-related cataract on blue-on-yellow perimetry. Invest Ophthalmol Vis Sci.

[20] Adams AJ, Rodic R, Husted R, Stamper R (1982). Spectral sensitivity and color discrimination changes in glaucoma and glaucoma-suspect patients. Invest Ophthalmol Vis Sci.

[21] Flammer J, Drance SM (1984). Correlation between color vision scores and quantitative perimetry in suspected glaucoma. Arch Ophthalmol.

[22] Hamill TR, Post RB, Johnson CA, Keltner JL (1984). Correlation of color vision deficits and observable changes in the optic disc in a population of ocular hypertensives. Arch Ophthalmol.

[23] Johnson CA, Adams AJ, Casson EJ, Brandt JD (1993). Progression of early glaucomatous visual field loss as detected by blue-on-yellow and standard white-on-white automated perimetry. Arch Ophthalmol.

[24] Sample PA, Taylor JD, Martinez GA, Lusky M, Weinreb RN (1993). Short-wavelength color visual fields in glaucoma suspects at risk. Am J Ophthalmol.

[25] Johnson CA, Adams AJ, Casson EJ, Brandt JD (1993). Blue-on-yellow perimetry can predict the development of glaucomatous visual field loss. Arch Ophthalmol.

[26] Sample PA, Martinez GA, Weinreb RN (1994). Short-wavelength automated perimetry without lens density testing. Am J Ophthalmol.

[27] Brusini P, Salvetat ML, Parisi L, Zeppieri M (2005). Probing glaucoma visual damage by rarebit perimetry. Br J Ophthalmol.

[28] Martin L, Wanger P (2004). New perimetric techniques: a comparison between rarebit and frequency doubling technology perimetry in normal subjects and glaucoma patients. J Glaucoma.

[29] Frisen L (2002). New, sensitive window on abnormal spatial vision: rarebit probing. Vision Res.

[30] Sample PA, Medeiros FA, Racette L, Pascual JP, Boden C, Zangwill LM (2006). Identifying glaucomatous vision loss with visual-function-specific perimetry in the diagnostic innovations in glaucoma study. Invest Ophthalmol Vis Sci.

[31] Heijl A, Leske MC, Bengtsson B, Bengtsson B, Hussein M (2003). Measuring visual field progression in the Early Manifest Glaucoma Trial. Acta Ophthalmol Scand.

[32] The advanced glaucoma intervention study (AGIS): 7 (2000). the relationship between control of intraocular pressure and visual field deterioration. Am J Ophthalmol.

[33] Keltner JL, Johnson CA, Quigg JM, Cello KE, Kass MA, Gordon MO (2000). Confirmation of visual field abnormalities in the Ocular Hypertension Treatment Study. Ocular Hypertension Treatment Study Group. Arch Ophthalmol.

[34] The Advanced Glaucoma Intervention Study (AGIS): 14 (2004). Distinguishing progression of glaucoma from visual field fluctuations. Ophthalmology.

[35] Bengtsson B, Heijl A (2008). A Visual Field Index for calculation of glaucoma rate of progression. Am J Ophthalmol.

